# LPI-IBNRA: Long Non-coding RNA-Protein Interaction Prediction Based on Improved Bipartite Network Recommender Algorithm

**DOI:** 10.3389/fgene.2019.00343

**Published:** 2019-04-18

**Authors:** Guobo Xie, Cuiming Wu, Yuping Sun, Zhiliang Fan, Jianghui Liu

**Affiliations:** ^1^School of Computers, Guangdong University of Technology, Guangzhou, China; ^2^Department of Emergency, The First Affiliated Hospital of Sun Yat-sen University, Guangzhou, China

**Keywords:** lncRNA, protein, interaction prediction, bipartite network, second-order correlation elimination

## Abstract

According to the latest research, lncRNAs (long non-coding RNAs) play a broad and important role in various biological processes by interacting with proteins. However, identifying whether proteins interact with a specific lncRNA through biological experimental methods is difficult, costly, and time-consuming. Thus, many bioinformatics computational methods have been proposed to predict lncRNA-protein interactions. In this paper, we proposed a novel approach called Long non-coding RNA-Protein Interaction Prediction based on Improved Bipartite Network Recommender Algorithm (LPI-IBNRA). In the proposed method, we implemented a two-round resource allocation and eliminated the second-order correlations appropriately on the bipartite network. Experimental results illustrate that LPI-IBNRA outperforms five previous methods, with the AUC values of 0.8932 in leave-one-out cross validation (LOOCV) and 0.8819 ± 0.0052 in 10-fold cross validation, respectively. In addition, case studies on four lncRNAs were carried out to show the predictive power of LPI-IBNRA.

## 1. Introduction

LncRNA, a class of ncRNAs (non-coding RNAs) of more than 200 nucleotides, that do not encode proteins, has gained increasing scientific interest in recent years (Jorge et al., 2012; Hajjari et al., 2016). Only 2% of RNAs in the human transcriptome can encode proteins while others, called ncRNAs, cannot. Note that most ncRNAs are lncRNAs. Compared to other ncRNAs, lncRNAs are longer and have complex secondary or higher-order structures (Bonasio and Shiekhattar, 2014), and their genes often have independent regulatory elements such as promoters and enhancers (Ulitsky and Bartel, 2013). There is increasing evidence that lncRNAs are related to the regulation of gene expression levels such as epigenetic regulation, transcriptional regulation, and multiple levels of post-transcriptional regulation (Sarah and Jeff, 2013), but only a few functions and mechanisms of lncRNA have been studied (Maarabouni et al., 2008; Lee et al., 2016). Moreover, interactions of lncRNAs with other molecules also have become hot spots in oncology research over the past years. The studies found that an important way for lncRNAs to function is by interacting with proteins (Khalil and Rinn, 2011). LncRNAs play a broad and important regulatory role in various processes such as tumorigenesis, cancer progression and metastasis by interacting with proteins. Thus, the prediction and identification of lncRNA-protein interactions can further reveal lncRNA-related functions and is beneficial for the study on the pathogenesis of complex diseases at the molecular level (Faghihi et al., 2008; Chen and Yan, 2013; Cui et al., 2013; Li et al., 2013; Chen et al., 2016a,c, 2017b, 2018c).

Numerous biological experimental methods were exploited to confirm protein-related RNAs (Ule et al., 2005; Galgano and Gerber, 2011; Zambelli and Pavesi, 2015). However, such experimental methods are laborious, time-consuming, and costly (Huang et al., 2012). Recently, various computational methods have been proposed to address the challenges in bioinformatics (He et al., 2018a,b; Zou et al., 2018), such as lncRNA-protein (Hu et al., 2017; Shen et al., 2019a,b), miRNA-disease (Chen and Huang, 2017; Chen et al., 2018a,b,d,e,f; Jiang et al., 2018a,b; Xie et al., 2018), drug-target (Chen et al., 2016b; Wang et al., 2017; Wu et al., 2018) and microbe-disease associations predictions (Chen et al., 2017a; Peng et al., 2018). The methods for inferring lncRNA-protein associations can roughly be classified into two types: the machine learning methods and the network-based methods. The so-called machine learning methods usually use the biological features of lncRNAs and proteins, and then employ a supervised classifier to identify whether proteins have potential interactions with a specific lncRNA (Zhan et al., 2018). For example, Bellucci et al. (2011) proposed to utilize secondary structure, hydrogen bonding and van der Waals contributions for feature integration, which has a beneficial effect for inferring the binding propensity of protein and ncRNA. Protein and lncRNA sequence information is utilized in Muppirala et al. (2011), with the employment of a support vector machine (SVM) and random forest (RF). Suresh et al. (2015) proposed an SVM-based method named RPI-Pred, which uses high-order 3D structural features and sequences of the lncRNA and protein. Hu et al. (2018) developed a method called HLPI-Ensembl, adopting the ensemble strategy based on extreme gradient boosting (XGB), SVM and RF. However, the main drawback of these methods is the insufficiency of negative samples of lncRNA-protein interactions. The lack of negative samples may cause the unstable performance of the supervised classifier. Moreover, selecting appropriate features to predict lncRNA-protein interactions is not an easy task.

Apart from the aforementioned methods, there are other approaches for potential lncRNA-protein interaction prediction, with the employment of network analysis algorithms. For instance, Li et al. (2015) presented a method called LPIHN, which constructs a heterogeneous network, and implements a random walk with restart on the heterogeneous network. In order to improve prediction performance, some recent network-based methods use recommender algorithms to infer lncRNA-protein interactions. For example, Ge et al. (2016) proposed a method called LPBNI, which only uses known lncRNA-protein interactions and implements the two-step propagation on a bipartite network. Zhao et al. (2018b) introduced an approach based on the bipartite network called LPI-BNPRA, which infers lncRNA-protein interactions by constructing bias ratings for lncRNAs and proteins, using agglomerative hierarchical clustering. By implementing two-round resource allocation on bipartite networks, these approaches achieved impressive results. But predictive validity of these investigations remains insufficient due to the existence of high-order correlations, which might have a negative effect on the lncRNA-protein interaction prediction. For example, the proteins directly correlated by the same lncRNA, could also be indirectly correlated by other media proteins, resulting in correlation redundancy. Properly eliminating the redundancy induced by the second-order correlation might further enhance the accuracy of the prediction. This inspired us to develop an effective network-based recommender algorithm for lncRNA-protein interaction prediction.

Motivated by the effectiveness of high-order correlation elimination in the study of Qiu et al. (2014), we propose a novel method named LPI-IBNRA for inferring new lncRNA-protein interactions. LPI-IBNRA uses known lncRNA-protein and protein-protein interactions, and lncRNA expression similarity, and then eliminates second-order correlations on the bipartite network appropriately to enhance the prediction accuracy. Compared with previous machine learning methods, our method does not require negative samples. Compared with many existing network-based methods (Ge et al., 2016; Zhao et al., 2018b), our method yields comparable or even better results due to second-order correlation elimination. Both 10-fold cross validation and LOOCV were carried out to assess the prediction ability of the proposed method. Experimental results illustrated that LPI-IBNRA outperformed five other methods by achieving higher AUC values. In addition, case studies on four lncRNAs further demonstrated the predictive power of LPI-IBNRA. Therefore, we conclude that LPI-IBNRA is feasible and effective for inferring potential lncRNA-protein interactions.

## 2. Materials and Methods

### 2.1. Human LncRNA-Protein Interactions

The known ncRNA-protein interaction dataset was downloaded from the NPInter v2.0 database (Yuan et al., 2014). We limited the organism to “Homo sapiens” and the type of ncRNAs to “NONCODE”, in order to filter ncRNAs and their interacting proteins. The lncRNAs were further filtered from these ncRNAs, through a human lncRNA dataset from the NONCODE 4.0 database (Xie et al., 2014). We deleted duplicate interactions. Considering the sample requirement of LOOCV, we removed the lncRNAs and proteins that have only one interaction. We then obtained 4796 distinct experimentally confirmed lncRNA-protein interactions, containing 26 proteins and 1105 lncRNAs. We denoted *np* as the number of known proteins, *nl* as the number of known lncRNAs, and matrix *I* ∈ ℝ^np*nl^ as the adjacency matrix of protein-lncRNA interactions. The interaction between protein *p*_*i*_ and lncRNA *l*_*j*_ could be denoted as follows:

(1) $$\begin{array}{l} I(p_{i},l_{j})=\{\begin{array}{ll} 1 & if\;p_{i}\;interacts\;with\;l_{j}\\ 0 & otherwise. \end{array} \end{array}$$

### 2.2. Protein-Protein Interaction Score Matrix and Similarity Matrix

Protein-protein interactions (PPI) were obtained from the STRING 9.1 database (Franceschini et al., 2013), which included weighted protein-protein interactions through co-expression data, genomic context predictions, automated text mining, and high-throughput lab experiments. We then deleted the redundant PPI data, and obtained 214 PPI data, and the corresponding interaction scores based on the known lncRNA-protein dataset. The symmetric matrix *AP* was denoted as an interaction score matrix based on PPI data, where *AP*(*p*_*i*_, *p*_*j*_) is the interaction score between proteins *p*_*i*_ and *p*_*j*_. *AP* could then be standardized as follows:

(2) $$\begin{array}{l} AP^{\prime }\left(p_{i},p_{j}\right)=\frac{AP\left(p_{i},p_{j}\right)}{\sqrt{R\left(p_{i}\right)R\left(p_{j}\right)}}, \end{array}$$

where *R*(*p*_*i*_) is the sum of the elements in *i*-row of *AP*.

Considering the hypothesis that similar proteins tend to exhibit a similar interaction and non-interaction pattern with lncRNAs (Zheng et al., 2017), we calculated the protein similarity with the utilization of Gaussian kernel interaction profiles. We denoted *X*(*p*_*i*_) as the *i*th row vector of matrix *I*, in which the nonzero values occur at the indices where the corresponding lncRNA have one interaction with a protein *p*_*i*_. Then the similarity between proteins *p*_*i*_ and *p*_*j*_ based on Gaussian kernel interaction profiles could be calculated as follows:

(3) $$\begin{array}{l} KP\left(p_{i},p_{j}\right)=exp\left(-\beta_{p}||X\left(p_{i}\right)-X\left(p_{j}\right)|{|}^{2}\right), \end{array}$$

where the adjustment coefficient β_*p*_ for the kernel bandwidth is defined as follows:

(4) $$\begin{array}{l} \beta_{p}=\beta_{p}^{\prime }/\left(\frac{1}{np}\overset{np}{\underset{i=1}{\sum }}||X\left(p_{i}\right)|{|}^{2}\right). \end{array}$$

### 2.3. LncRNA-LncRNA Similarity Matrix

LncRNA expression profiles were downloaded from the NONCODE 4.0 database (Xie et al., 2014). After removing the superfluous data, we obtained the expression profiles of 1,105 lncRNAs in 24 human tissues or cell types. Then the Pearson correlation coefficient (PPC) was applied for the calculation of lncRNA expression similarity between each pair of lncRNA expression profiles (Wang et al., 2010; Ganegoda et al., 2013; Tang et al., 2014). We denoted *E*(*i*) = {*e*_*i*1_, *e*_*i*2_, …, *e*_*i*24_} and *E*(*j*) = {*e*_*j*1_, *e*_*j*2_, …, *e*_*j*24_} as the expression profiles of *l*_*i*_ and *l*_*j*_. The expression similarity *AL*(*l*_*i*_, *l*_*j*_) between lncRNAs *l*_*i*_ and *l*_*j*_ was calculated as follows:

(5) $$\begin{array}{l} AL\left(l_{i},l_{j}\right)=|\frac{cov\left(E\left(i\right),E\left(j\right)\right)}{\sigma_{E\left(i\right)}\times \sigma_{E\left(j\right)}}|, \end{array}$$

where *AL*(*l*_*i*_, *l*_*j*_) denotes the absolute value of PCC between *l*_*i*_ and *l*_*j*_, *cov*(*E*(*i*), *E*(*j*)) is the covariance between *E*(*i*) and *E*(*j*), σ_*E*(*i*)_ and σ_*E*(*j*)_ are standard deviations of *E*(*i*) and *E*(*j*), respectively.

We denoted *X*(*p*_*i*_) as the *i*th column vector of matrix *I*, in which the nonzero values occur at the indices where the corresponding protein has one interaction with the lncRNA *l*_*i*_. Similar to the aforementioned protein case, the Gaussian interaction profile kernel similarity for lncRNAs could be computed as follows:

(6) $$\begin{array}{l} KL\left(l_{i},l_{j}\right)=exp\left(-\beta_{l}||X\left(l_{i}\right)-X\left(l_{j}\right)|{|}^{2}\right), \end{array}$$

where

(7) $$\begin{array}{l} \beta_{l}=\beta_{l}^{\prime }/\left(\frac{1}{nl}\overset{nl}{\underset{i=1}{\sum }}||X\left(l_{i}\right)|{|}^{2}\right). \end{array}$$

### 2.4. Integrated Similarity Matrix for Proteins and LncRNAs

Note that the Gaussian interaction profile kernel similarity is an association information-based measurement, which can be utilized to complement protein-protein interactions and lncRNA expression similarity. Motivated by the study of Chen (2015), we constructed the integrated protein similarity matrix *Sim*^*P*^ and integrated the lncRNA similarity matrix *Sim*^*L*^ as follows:

(8) $$\begin{array}{l} Sim^{P}(p_{i},p_{j})=\{\begin{array}{ll} \frac{AP^{\prime }(p_{i},p_{j})+KP(p_{i},p_{j})}{2} & if\;AP^{\prime }(p_{i},p_{j})\neq 0\\ KP(p_{i},p_{j}) & otherwise, \end{array} \end{array}$$

(9) $$\begin{array}{l} Sim^{L}\left(l_{i},l_{j}\right)=\frac{AL\left(l_{i},l_{j}\right)+KL\left(l_{i},l_{j}\right)}{2}. \end{array}$$

### 2.5. LPI-IBNRA

**Figure 1:**
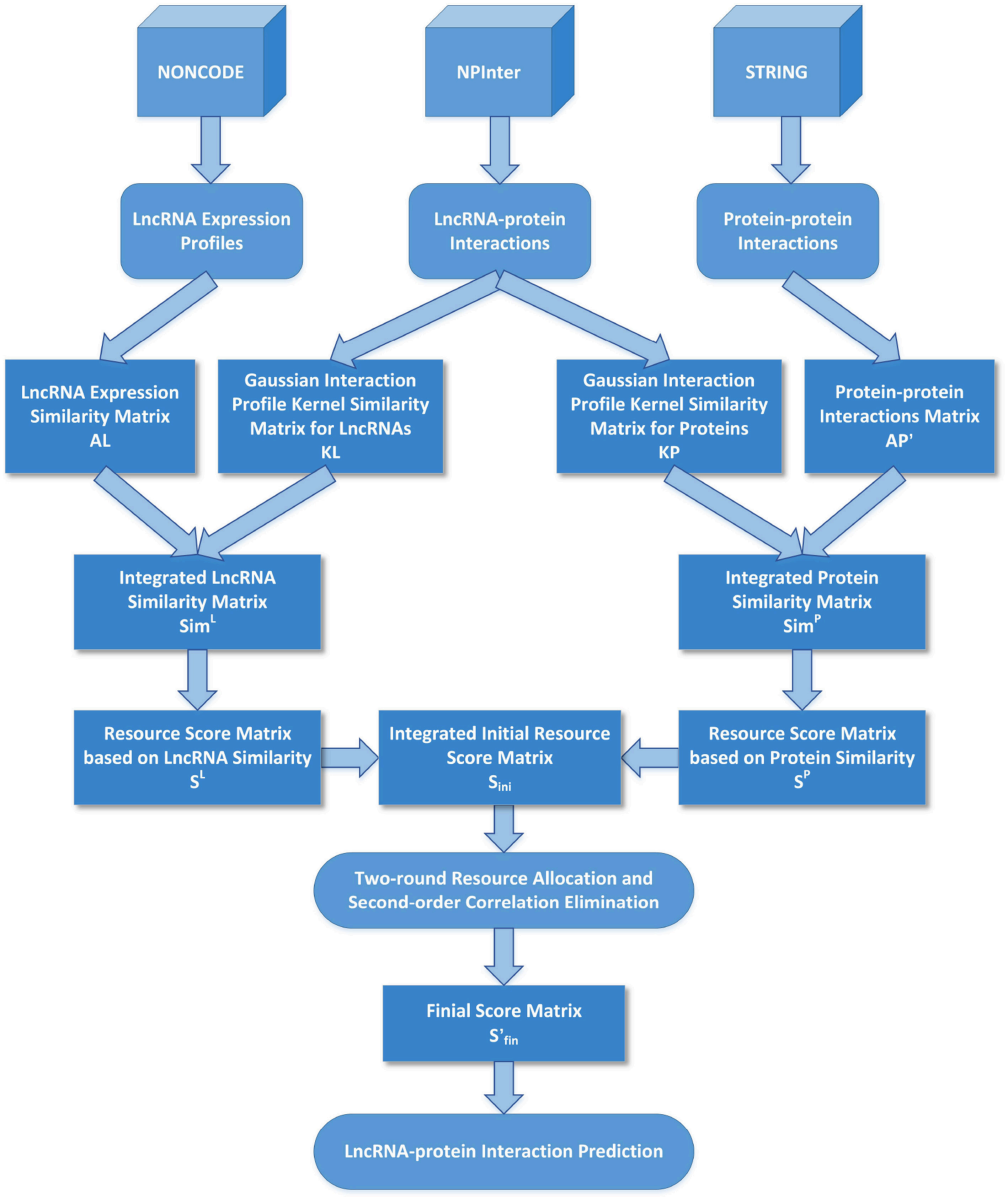
The flowchart of LPI-IBNRA method.

The flow chart of LPI-IBNRA is shown in Figure 1. At first, we denoted *S*^*P*^ ∈ ℝ^np*nl^ as the resource score matrix based on protein similarity, *S*^*L*^ ∈ ℝ^np*nl^ as the one based on lncRNA similarity. These two matrices were computed as follows:

(10) $$\begin{array}{l} S^{P}(p_{i},l_{j})=\{\begin{array}{ll} \frac{\sum_{k=1}^{np}Sim^{P}(p_{i},p_{k})I(p_{k},l_{j})}{\sum_{k=1}^{np}Sim^{P}(p_{i},p_{k})} & if\;I(p_{i},l_{j})=1\\ 0 & otherwise, \end{array} \end{array}$$

(11) $$\begin{array}{l} S^{L}(p_{i},l_{j})=\{\begin{array}{ll} \frac{\sum_{k=1}^{nl}I(p_{i},l_{k})Sim^{L}(l_{k},l_{j})}{\sum_{k=1}^{nl}Sim^{L}(l_{k},l_{i})} & if\;I(p_{i},l_{j})=1\\ 0 & otherwise, \end{array} \end{array}$$

where $S^{P}\left(p_{i},l_{j}\right)$ represents the score between protein *p*_*i*_ and lncRNA *l*_*j*_ based on protein similarity, and $S^{L}\left(p_{i},l_{j}\right)$ represents the score between protein *p*_*i*_ and lncRNA *l*_*j*_ based on lncRNA similarity.

Then the integrated resource score matrix was initialized as the weighted sum of *S*^*P*^ and *S*^*L*^ as follows:

(12) $$\begin{array}{l} S_{ini}=\gamma S^{P}+\left(1-\gamma \right)S^{L}, \end{array}$$

where parameter γ ∈ [0, 1] is a scalar controlling the relative contributions of protein similarity and lncRNA similarity in *S*_*ini*_. Following the general setting, we set the parameter γ = 0.5 in this paper, making *S*^*P*^ and *S*^*L*^ equally weighted.

The final score matrix can be obtained by updating the *S*_*ini*_ column by column. In other words, the calculation process can be partitioned into *nl* runs, each of which corresponds to a specific lncRNA. Thus, at the beginning of the *k*th run, the score for protein *p*_*i*_ interacting with the given lncRNA *l*_*k*_ can be initialized as follows:

(13) $$\begin{array}{l} s_{0}\left(p_{i}\right)=S_{ini}\left(p_{i},l_{k}\right). \end{array}$$

Then the 1st-round of our allocation model is to allocate the score of the lncRNA *l*_*k*_ from the protein *p*_*i*_, which can be calculated as follows:

(14) $$\begin{array}{l} s_{1}\left(p_{i},l_{k}\right)=\frac{S_{ini}\left(p_{i},l_{k}\right)s_{0}\left(p_{i}\right)}{d\left(p_{i}\right)}, \end{array}$$

where $d\left(p_{i}\right)=\overset{nl}{\underset{x=1}{\sum }}S_{ini}\left(p_{i},l_{x}\right)$ is obtained by a summing operation over all initial scores from lncRNAs interacting with protein *p*_*i*_.

The score of lncRNA *l*_*k*_ can be obtained by summing scores over all proteins connected with *l*_*k*_:

(15) $$\begin{array}{l} s_{1}\left(l_{k}\right)=\overset{np}{\underset{j=1}{\sum }}s_{1}\left(p_{j},l_{k}\right)=\overset{np}{\underset{j=1}{\sum }}\frac{S_{ini}\left(p_{j},l_{k}\right)s_{0}\left(p_{j}\right)}{d\left(p_{j}\right)}. \end{array}$$

In the 2nd-round, resource scores were allocated in a similar way as the first round. The score allocated from the lncRNA *l*_*k*_ to the protein *p*_*i*_ was calculated as follows:

(16) $$\begin{array}{l} s_{2}\left(p_{i},l_{k}\right)=\frac{S_{ini}\left(p_{i},l_{k}\right)s_{1}\left(l_{k}\right)}{d\left(l_{k}\right)}, \end{array}$$

where $d\left(l_{k}\right)=\overset{np}{\underset{y=1}{\sum }}S_{ini}\left(p_{y},l_{k}\right)$ is the sum of initial scores from all proteins interacting with lncRNA *l*_*k*_.

The score of protein *p*_*i*_ was allocated from all lncRNAs that interacted with *p*_*i*_ as follows:

(17) $$\begin{array}{l} s_{2}\left(p_{i}\right)=\overset{nl}{\underset{k=1}{\sum }}\frac{S_{ini}\left(p_{i},l_{k}\right)s_{1}\left(l_{k}\right)}{d\left(l_{k}\right)}=\overset{nl}{\underset{k=1}{\sum }}\frac{S_{ini}\left(p_{i},l_{k}\right)}{d\left(l_{k}\right)}\overset{np}{\underset{j=1}{\sum }}\frac{S_{ini}\left(p_{j},l_{k}\right)s_{0}\left(p_{j}\right)}{d\left(p_{j}\right)}. \end{array}$$

**Figure 2:**
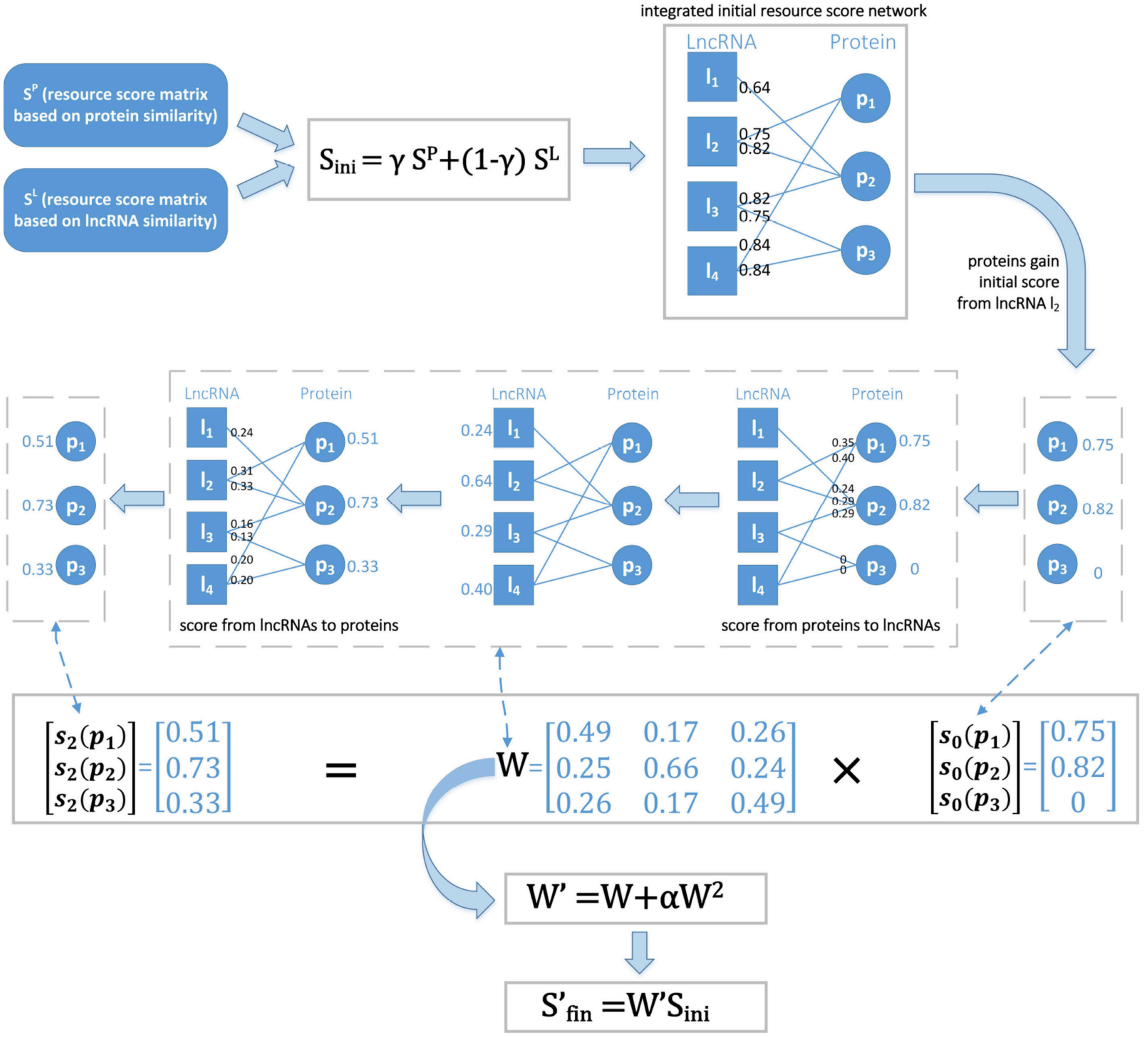
The basic idea of LPI-IBNRA. First, two resource score matrices which are computed based on protein similarity and lncRNA similarity, respectively, are combined to construct the initial integrated resource score network. Secondly, each protein gains its initial score from a specific lncRNA. Next, in two-round resource allocation, the score is allocated from proteins to lncRNAs, and then propagated back to proteins. Finally, the weight matrix is optimized by second-order correlation eliminations to obtain the final scores of proteins.

As described from Equation (13) to (17), we first initialized the score of protein *p*_*i*_ from the given lncRNA *l*_*k*_ and then updated it by a two-round resource allocation. An example is given in Figure 2. We defined $S_{fin}\in {\mathbb{R}}^{np*nl}$ as the final resource score matrix, which can be represented as follows:

(18) $$\begin{array}{l} S_{fin}\left(p_{i},l_{k}\right)=s_{2}\left(p_{i}\right). \end{array}$$

*S* can also be computed in a vectorized form as:

(19) $$\begin{array}{l} \vec{S_{fin}}=W\vec{S_{ini}}, \end{array}$$

where $\vec{S_{fin}}$ is a column vector of *S*_*fin*_, $\vec{S_{ini}}$ is a column vector of *S*_*ini*_, and *W* ∈ ℝ^np*np^ is the weight matrix. Then Equation (17) can also be represented as:

(20) $$\begin{array}{l} s_{2}\left(p_{i}\right)=\overset{np}{\underset{j=1}{\sum }}W\left(p_{i},p_{j}\right)s_{0}\left(p_{j}\right), \end{array}$$

where

(21) $$\begin{array}{l} W\left(p_{i},p_{j}\right)=\frac{1}{d\left(p_{j}\right)}\overset{nl}{\underset{k=1}{\sum }}\frac{S_{ini}\left(p_{i},l_{k}\right)S_{ini}\left(p_{j},l_{k}\right)}{d\left(l_{k}\right)}. \end{array}$$

In the lncRNA-protein interaction network, the proteins interacting with the same lncRNA are considered to be directly correlated, i.e., having the low-order correlation, while higher-order correlations between these proteins might also arise from indirect associations. Such high-order correlations might have a negative effect on the lncRNA-protein interaction prediction. Based on the studies of Zhou et al. (2009) and Liu et al. (2010), we eliminated second-order correlations in an appropriate way to further enhance the accuracy of the prediction:

(22) $$\begin{array}{l} W^{\prime }=W+\alpha W^{2}, \end{array}$$

where the parameter α ∈ (−1, 0). The final score matrix for inferring potential lncRNA-protein interactions can then be calculated as follows:

(23) $$\begin{array}{l} S_{fin}^{\prime }=W^{\prime }S_{ini}. \end{array}$$

After the calculations, we can recommend proteins to the given lncRNA *l*_*k*_ in descending order by the *k*th column of $S_{fin}^{\prime }$.

### 2.6. Performance Evaluation

We evaluated the classification performance of the proposed LPI-IBNRA method by applying two types of classification schemes, i.e., LOOCV and 10-fold cross validation. The performance of LPI-IBNRA was evaluated in terms of several widely-used indicators, including precision (PRE), sensitivity (SEN), accuracy (ACC), F1 score, and Matthews correlation coefficient (MCC), expressed as follows:

(24) $$\begin{array}{l} PRE=\frac{TP}{TP+FP}, \end{array}$$

(25) $$\begin{array}{l} SEN=\frac{TP}{TP+FN}, \end{array}$$

(26) $$\begin{array}{l} ACC=\frac{TP+TN}{TP+TN+FP+FN}, \end{array}$$

(27) $$\begin{array}{l} F1\;Score=\frac{2\times TP}{2\times TP+FP+FN}=2\times \frac{PRE\times SEN}{PRE+SEN}, \end{array}$$

(28) $$\begin{array}{l} MCC=\frac{\left(TP+TN\right)-\left(FP+FN\right)}{\sqrt{\left(TN+FN\right)\times \left(TN+FP\right)\times \left(TP+FN\right)\times \left(TP+FP\right)}}. \end{array}$$

where TP, TN, FP, and FN count the number of true positives, true negatives, false positives, and false negatives, respectively.

As a popular method for performance evaluation, the receiver operating characteristic (ROC) curve was also utilized in our experiments. The area under the ROC curve (AUC) = 1 indicates perfect performance, while AUC = 0.5 indicates random performance. The precision-recall curve (PR curve) and the area under the PR curve (AUPR) are also used to reduce the negative influence of false positive data on the method performance. The larger the AUC and AUPR is, the better performance the evaluated method has.

## 3. Results

### 3.1. Comparison With Other Methods

**Figure 3:**
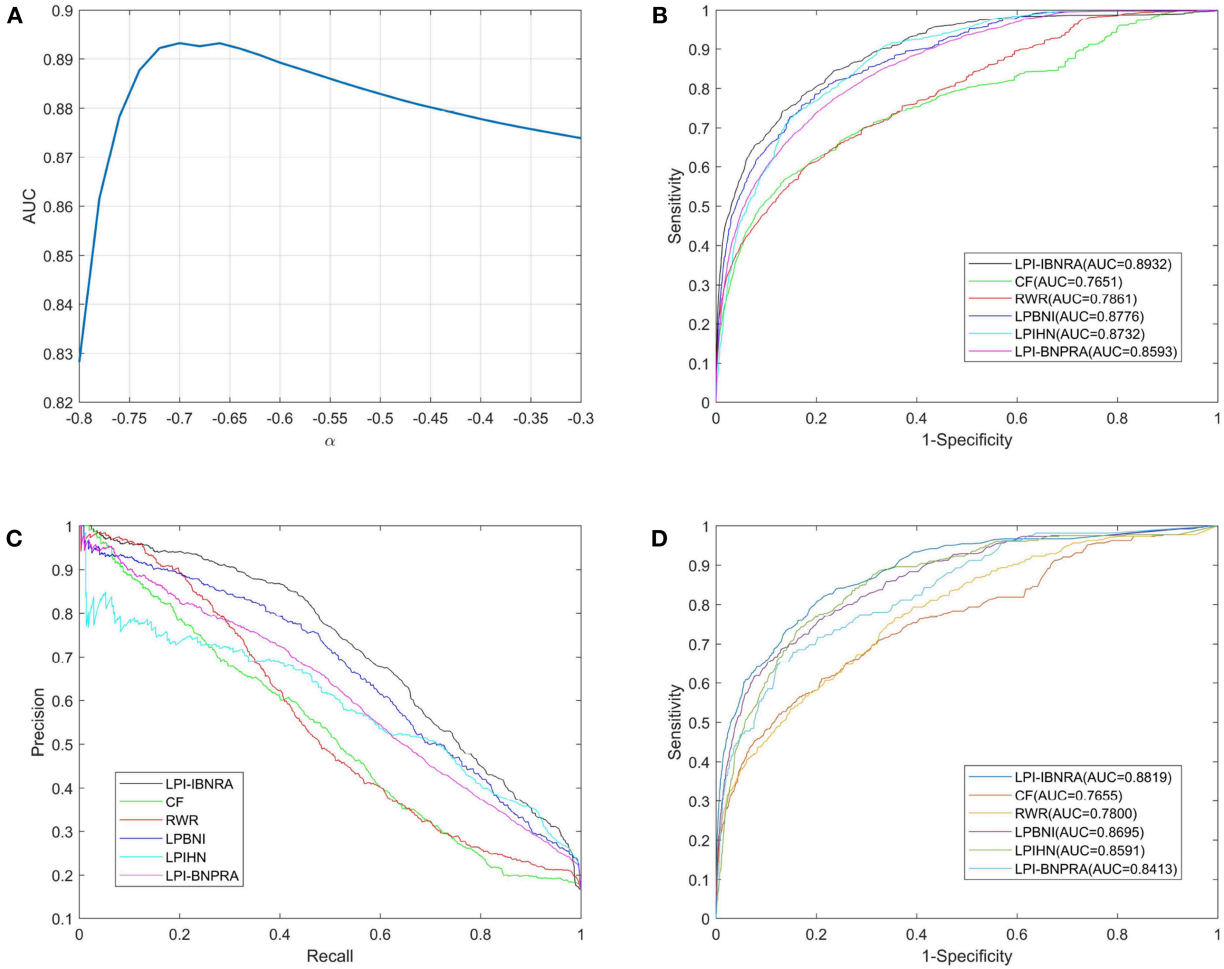
**(A)**The AUC values of LPI-IBNRA method with different values of α. **(B)** ROC curves of lncRNA-protein interaction predictions by all compared methods in LOOCV. **(C)** Precision-recall curves of all compared methods. **(D)** ROC curves of lncRNA-protein interaction predictions by all compared methods in 10-fold cross validation.

We used the aforementioned 4,796 known human lncRNA-protein interactions to carry out the above-mentioned two cross validation schemes. In each LOOCV trial, each known lncRNA-protein interaction was used as a test sample while the rest were used as training samples. To analyze the influence of parameter α on the performance of LPI-IBNRA, we applied LOOCV for the selection of parameter α. As shown in Figure 3A, the performance of LPI-IBNRA drops a lot when α is smaller than –0.70. When α is larger than –0.70, the performance of LPI-IBNRA decreases slightly. Thus, the parameter α is set to –0.70 due to the optimal performance.

Five previous approaches were used for comparison in the experiments, including collaborative filtering (CF), random walk with restart (RWR), LPBNI, LPIHN, and LPI-BNPRA. LPBNI, LPIHN, and LPI-BNPRA are network-based methods that infer potential lncRNA-protein interactions, while CF and RWR have been used as benchmark methods in Ge et al. (2016) and Wen et al. (2017). RWR is often utilized as a powerful tool for network-based methods to forecast association (Zhao et al., 2018a,c; Zhu et al., 2018), while CF is a well-known recommender algorithm which can infer the information from similar neighborhoods (Fu et al., 2014; Zeng et al., 2017). In our experiments, RWR was implemented to make predictions based on the protein-protein similarity network, while a simple version of the CF algorithm was adopted to calculate the prediction scores between lncRNAs and proteins.

Here, we reproduced these methods on the same dataset by ourselves. See Figures 3B,C and Table 1 for the results of LOOCV. We can see from Figure 3B that our proposed method achieved an AUC of 0.8932, which exhibited a considerable improvement over the five previous methods (i.e., 12.81% for CF, 10.71% for RWR, 1.56% for LPBNI, 2.00% for LPIHN and 3.39% for LPI-BNPRA). In addition, the comparison of these methods, in terms of precision vs. recall, is presented in Figure 3C. It can be seen that LPI-IBNRA almost achieved a higher precision than the other methods at every recall value. Moreover, LPI-IBNRA outperformed the other methods in terms of AUPR, PRE, SEN, ACC, F1 score and MCC, which is presented in Table 1. As shown in Figure 3D, in 10-fold cross validation, LPI-IBNRA achieved an AUC of 0.8819 ± 0.0052 and was superior to the comparison methods, including CF (0.7655 ± 0.0069), RWR (0.7800 ± 0.0076), LPBNI (0.8695 ± 0.0047), LPIHN (0.8591 ± 0.0044), and LPI-BNPRA (0.8413 ± 0.0351).

**Table 1:** Performance evaluation of all compared methods in LOOCV in terms of several widely-used indicators.

**Methods**	**AUC**	**AUPR**	**PRE**	**SEN**	**ACC**	**F1-score**	**MCC**
LPI-IBNRA	0.8932	0.7098	0.8778	0.3580	0.8845	0.5273	0.5152
CF	0.7651	0.5249	0.7941	0.1922	0.8568	0.3225	0.3452
RWR	0.7861	0.5500	0.8316	0.2460	0.8658	0.3949	0.4063
LPBNI	0.8776	0.6624	0.8526	0.2882	0.8729	0.4474	0.4496
LPIHN	0.8732	0.5892	0.7518	0.1522	0.8501	0.2642	0.2933
LPI-BNPRA	0.8593	0.6148	0.8200	0.2271	0.8627	0.3701	0.3855

The aforementioned results indicate that in both LOOCV and 10-fold cross evaluation, LPI-IBNRA outperforms other methods in terms of the AUC values. The outstanding performance of LPI-IBNRA demonstrates its stable and satisfying abilities in inferring potential lncRNA-protein interactions. The superior performance of the proposed method could be attributed to second-order correlation elimination, which is more suitable for our task and can lead to better prediction performance.

### 3.2. Case Studies

In addition, four case studies have been carried out to further evaluate the effectiveness of LPI-IBNRA. The interactions in our benchmark dataset were obtained in NPInter v2.0 which was established in 2013. NPInter was then upgraded to NPInter v3.0 in 2016 (Hao et al., 2016), which includes newly discovered lncRNA-protein interactions. Thus, we predicted novel lncRNA-protein interactions based on known interactions in the benchmark dataset, then confirmed our predictions in NPInter v3.0. For each lncRNA, the proteins ranked within the top 5 were considered as potential proteins that interact with the given lncRNA. Case studies were carried out on four lncRNAs, including lncRNA DLEU2, CRHR1-1T1, LRRC75A-AS1 and SNHG5.

Table 2 shows the prediction results and whether there were confirmations for these lncRNAs. It indicates that five (DLEU2), five (CRHR1-1T1), five (LRRC75A-AS1), and four (SNHG5) out of the top five predicted lncRNA-interacted proteins, were confirmed by NPInter v3.0. The rankings of these lncRNA-protein interactions in other benchmark method predictions are also listed in Table 2. It can be observed that several novel interactions did not have high rankings in the predictions of other methods, and these interactions are likely to be ignored by these methods. Therefore, LPI-IBNRA has great potential to predict new lncRNA-protein interactions.

**Table 2:** The top five ranked proteins for lncRNA DLEU2, CRHR1-1T1, LRRC75A-AS1, and SNHG5.

**LncRNA**	**Protein**	**Confirmed?**	**Rank**	**CF**	**RWR**	**LPBNI**	**LPIHN**	**LPI-BNPRA**
DLEU2	ENSP00000290341	Confirmed	1	7	1	1	3	1
DLEU2	ENSP00000258729	Confirmed	2	17	2	2	1	6
DLEU2	ENSP00000381031	Confirmed	3	1	6	3	5	7
DLEU2	ENSP00000371634	Confirmed	4	16	3	6	2	8
DLEU2	ENSP00000254108	Confirmed	5	10	4	4	4	2
CRHR1-IT1	ENSP00000254108	Confirmed	1	11	4	3	2	2
CRHR1-IT1	ENSP00000240185	Confirmed	2	2	9	2	11	7
CRHR1-IT1	ENSP00000350028	Confirmed	3	1	10	8	12	10
CRHR1-IT1	ENSP00000381031	Confirmed	4	12	11	9	5	8
CRHR1-IT1	ENSP00000220592	Confirmed	5	5	1	4	10	6
LRRC75A-AS1	ENSP00000385269	Confirmed	1	6	1	1	1	1
LRRC75A-AS1	ENSP00000254108	Confirmed	2	7	2	2	2	2
LRRC75A-AS1	ENSP00000381031	Confirmed	3	8	4	3	6	3
LRRC75A-AS1	ENSP00000350028	Confirmed	4	9	6	7	12	9
LRRC75A-AS1	ENSP00000258962	Confirmed	5	1	10	5	16	5
SNHG5	ENSP00000290341	Confirmed	1	1	1	1	4	1
SNHG5	ENSP00000350028	Confirmed	2	2	10	4	6	3
SNHG5	ENSP00000240185	Confirmed	3	7	11	3	13	4
SNHG5	ENSP00000254108		4	3	2	2	1	2
SNHG5	ENSP00000381031	Confirmed	5	4	8	5	10	6

## 4. Discussion and Conclusion

In this article, we proposed a novel method LPI-IBNRA for predicting lncRNA-protein interactions, based on the known lncRNA-protein interactions, lncRNA expression similarity and protein-protein interactions. We integrated the known interactions and similarity as the initial resource scores for a two-round resource allocation of a bipartite network recommendation. Furthermore, we optimized the weight matrix by eliminating second-order correlations appropriately, to obtain the final result of lncRNA-protein interaction prediction. We finally acquired gratifying and reliable prediction performance in LOOCV, 10-fold cross evaluation and case studies. Thus, we believe that LPI-IBNRA can make reliable predictions and might guide future experimental studies on lncRNA-protein interactions.

LPI-IBNRA has the following improvements over several previous methods in predicting lncRNA-protein interactions. First, with the employment of the bipartite network recommender algorithm, we utilized the known lncRNA-protein interactions to construct a bipartite network between lncRNAs and proteins, and then allocated the resource scores via interaction edges between lncRNA nodes and protein nodes. Therefore, the negative sample set is not required in our methods. Second, we assigned weights to each edge on the bipartite network, which is distinct from most former bipartite network methods. Thus, the resource scores would not be evenly distributed during the resource allocation process. Finally, we eliminated second-order correlations on the bipartite network appropriately, to enhance prediction accuracy.

Although impressive results have been achieved, there is still much room for improvement in our method. At first, though known lncRNA-protein interactions have been more than before, it is still very difficult for the proposed method to obtain adequate results based on the prediction. Moreover, as the resource allocation of the bipartite network recommendation algorithm is based on known lncRNA-protein interactions, LPI-IBNRA is not suitable to predict interactions of lncRNAs without any known interacted protein.

## Author Contributions

GX designed the experiments. CW and ZF performed the experiments. GX, CW, YS, ZF, and JL conceived the project and analyzed the data. CW and YS wrote the manuscript and all authors contributed to the writing.

### Conflict of Interest Statement

The authors declare that the research was conducted in the absence of any commercial or financial relationships that could be construed as a potential conflict of interest.

## References

[B1] Bellucci M. Agostini F. Masin M. Tartaglia G. G. (2011). Predicting protein associations with long noncoding rnas. Nat. Methods 8, 444–445. 10.1038/nmeth.1611 21623348

[B2] Bonasio R. Shiekhattar R. (2014). Regulation of transcription by long noncoding rnas. Ann. Rev. Genet. 48, 433–455. 10.1146/annurev-genet-120213-092323 25251851 PMC4285387

[B3] Chen X. (2015). Katzlda: katz measure for the lncrna-disease association prediction. Sci. Rep. 5:16840. 26577439 10.1038/srep16840 PMC4649494

[B4] Chen X. Guan N. N. Sun Y. Z. Li J. Q. Qu J. (2018a). Microrna-small molecule association identification: from experimental results to computational models. Brief. Bioinformat. bby098. 10.1093/bib/bby098 30325405

[B5] Chen X. Huang L. (2017). Lrsslmda: laplacian regularized sparse subspace learning for mirna-disease association prediction. PLoS Comput. Biol. 13:e1005912. 10.1371/journal.pcbi.1005912 29253885 PMC5749861

[B6] Chen X. Huang L. Xie D. Zhao Q. (2018b). Egbmmda: extreme gradient boosting machine for mirna-disease association prediction. Cell Death Disease 9:3. 10.1038/s41419-017-0003-x 29305594 PMC5849212

[B7] Chen X. Huang Y. A. You Z. H. Yan G. Y. Wang X. S. (2017a). A novel approach based on katz measure to predict associations of human microbiota with non-infectious diseases. Bioinformatics 33, 733–739. 10.1093/bioinformatics/btw715 28025197

[B8] Chen X. Sun Y. Z. Guan N. Qu J. Li J. Q. (2018c). Computational models for lncrna function prediction and functional similarity calculation. Brief. Fun. Genom. 18, 58–82. 10.1093/bfgp/ely031 30247501

[B9] Chen X. Sun Y. Z. Liu H. Zhang L. Li J. Q. Meng J. (2017b). Rna methylation and diseases: experimental results, databases, web servers and computational models. Brief. Bioinform. bbx142. 10.1093/bib/bbx142 29165544

[B10] Chen X. Wang L. Qu J. Guan N. N. Li J. Q. (2018d). Predicting miRNA-disease association based on inductive matrix completion. Bioinformatics 34, 4256–4265. 10.1093/bioinformatics/bty503 29939227

[B11] Chen X. Xie D. Wang L. Zhao Q. Liu H. (2018e). Bnpmda: bipartite network projection for mirna-disease association prediction. Bioinformatics 34, 3178–3186. 10.1093/bioinformatics/bty333 29701758

[B12] Chen X. Yan C. G. C. Zhang X. You Z. (2016a). Long non-coding rnas and complex diseases: from experimental results to computational models. Brief. Bioinform. 18, 558–576. 10.1093/bib/bbw060 27345524 PMC5862301

[B13] Chen X. Yan C. C. Zhang X. Zhang X. Dai F. Yin J.. (2016b). Drug-target interaction prediction: databases, web servers and computational models. Brief. Bioinform. 17, 696–712. 10.1093/bib/bbv066 26283676

[B14] Chen X. Yan G. Y. (2013). Novel human lncrna-disease association inference based on lncrna expression profiles. Bioinformatics 29, 2617–2624. 10.1093/bioinformatics/btt426 24002109

[B15] Chen X. Yin J. Qu J. Huang L. (2018f). Mdhgi: matrix decomposition and heterogeneous graph inference for mirna-disease association prediction. PLoS Comput. Biol. 14:e1006418. 10.1371/journal.pcbi.1006418 30142158 PMC6126877

[B16] Chen X. You Z. H. Yan G. Y. Gong D. W. (2016c). Irwrlda: improved random walk with restart for lncrna-disease association prediction. Oncotarget 7, 57919–57931. 10.18632/oncotarget.11141 27517318 PMC5295400

[B17] Cui Z. Ren S. Lu J. Wang F. Xu W. D. Sun Y.. (2013). The prostate cancer-up-regulated long noncoding rna plncrna-1 modulates apoptosis and proliferation through reciprocal regulation of androgen receptor. Urol. Oncol. Sem. Original Investig. 31, 1117–1123. 10.1016/j.urolonc.2011.11.030 22264502

[B18] Faghihi M. A. Modarresi F. Khalil A. M. Wood D. E. Sahagan B. G. Morgan T. E.. (2008). Expression of a noncoding rna is elevated in alzheimer's disease and drives rapid feed-forward regulation of β-secretase. Nat. Med. 14, 723–730. 10.1038/nm1784 18587408 PMC2826895

[B19] Franceschini A. Szklarczyk D. Frankild S. Kuhn M. Simonovic M. Roth A.. (2013). String v9.1: protein-protein interaction networks, with increased coverage and integration. Nucleic Acids Res. 41, D808–D815. 10.1093/nar/gks1094 23203871 PMC3531103

[B20] Fu Y. Liu Q. Cui Z. (2014). A collaborative recommend algorithm based on bipartite community. Sci. World J. 2014, 1–14. 10.1155/2014/295931 24955393 PMC4009125

[B21] Galgano A. Gerber A. P. (2011). Rna-binding protein immunopurification-microarray (rip-chip) analysis to profile localized rnas. Methods Mol. Biol. 714, 369–385. 10.1007/978-1-61779-005-8-23 21431753

[B22] Ganegoda G. U. Wang J. X. Wu F. X. Li M. (2013). Prioritization of candidate genes based on disease similarity and protein's proximity in ppi networks, in IEEE International Conference on Bioinformatics and Biomedicine, Shanghai, 103–108.

[B23] Ge M. Li A. Wang M. (2016). A bipartite network-based method for prediction of long non-coding rna-protein interactions. Genom. Proteom. Bioinform. 14, 62–71. 10.1016/j.gpb.2016.01.004 26917505 PMC4792848

[B24] Hajjari M. Mowla S. J. Faghihi M. A. (2016). Editorial: molecular function and regulation of non-coding rnas in multifactorial diseases. Front. Genet. 7:9. 10.3389/fgene.2016.00009 26925093 PMC4760206

[B25] Hao Y. Wu W. Li H. Yuan J. Luo J. Zhao Y.. (2016). Npinter v3.0: an upgraded database of noncoding rna-associated interactions. Database 2016:baw057. 10.1093/database/baw057 27087310 PMC4834207

[B26] He W. Jia C. Duan Y. Zou Q. (2018a). 70propred: a predictor for discovering sigma70 promoters based on combining multiple features. BMC Syst. Biol. 12:44. 10.1186/s12918-018-0570-1 29745856 PMC5998878

[B27] He W. Ju Y. Zeng X. Liu X. Zou Q. (2018b). Sc-ncdnapred: a sequence-based predictor for identifying non-coding dna in saccharomyces cerevisiae. Front. Microbiol. 9:2174. 10.3389/fmicb.2018.02174 30258427 PMC6144933

[B28] Hu H. Zhang L. Ai H. Zhang H. Fan Y. T. Zhao Q.. (2018). Hlpi-ensemble: prediction of human lncrna-protein interactions based on ensemble strategy. RNA Biol. 15, 797–806. 10.1080/15476286.2018.1457935 29583068 PMC6152435

[B29] Hu H. Zhu C. Ai H. Zhang L. Zhao J. Zhao Q.. (2017). Lpi-etslp: lncrna-protein interaction prediction using eigenvalue transformation-based semi-supervised link prediction. Mol. Biosyst. 13, 1781–1787. 10.1039/C7MB00290D 28702594

[B30] Huang Y. Y. Yang X. F. Li H. T. Ji X. F. Cheng H. L. Zhao Y. J. (2012). Protein-rna interaction interface prediction and design. Acta Phys. Chim. Sin. 28, 2390–2400. 10.3866/PKU.WHXB201209111

[B31] Jiang L. Ding Y. Tang J. Guo F. (2018a). Mda-skf: similarity kernel fusion for accurately discovering mirna-disease association. Front. Genet. 9:618. 10.3389/fgene.2018.00618 30619454 PMC6295467

[B32] Jiang L. Xiao Y. Ding Y. Tang J. Guo F. (2018b). Fkl-spa-laprls: an accurate method for identifying human microrna-disease association. BMC Genom. 19:911. 10.1186/s12864-018-5273-x 30598109 PMC6311941

[B33] Jorge N. A. Ferreira C. G. Passetti F. (2012). Bioinformatics of cancer ncrna in high throughput sequencing: present state and challenges. Front. Genet. 3:287. 10.3389/fgene.2012.00287 23251139 PMC3523245

[B34] Khalil A. M. Rinn J. L. (2011). Rna-protein interactions in human health and disease. Sem. Cell Dev. Biol. 22, 359–365. 10.1016/j.semcdb.2011.02.016 21333748 PMC3184770

[B35] Lee S. Kopp F. Chang T. C. Sataluri A. Chen B. Sivakumar S.. (2016). Noncoding rna norad regulates genomic stability by sequestering pumilio proteins. Cell 164, 69–80. 10.1016/j.cell.2015.12.017 26724866 PMC4715682

[B36] Li A. Ge M. Zhang Y. Peng C. Wang M. H. (2015). Predicting long noncoding rna and protein interactions using heterogeneous network model. Biomed Res. Int. 2015, 1–11. 10.1155/2015/671950 26839884 PMC4709602

[B37] Li J. Xuan Z. Liu C. N. (2013). Long non-coding rnas and complex human diseases. Int. J. Mol. Sci. 14, 18790–18808. 10.3390/ijms140918790 24036441 PMC3794807

[B38] Liu J. G. Zhou T. Che H. A. Wang B. H. Zhang Y. C. (2010). Effects of high-order correlations on personalized recommendations for bipartite networks. Phys. A Statis. Mech. Appl. 389, 881–886. 10.1016/j.physa.2009.10.027

[B39] Maarabouni M. Pickard M. Hedge V. L. Farzaneh F. Williams G. (2008). Gas5, a non-protein-coding rna, controls apoptosis and is downregulated in breast cancer. Oncogene 28, 195–208. 10.1038/onc.2008.373 18836484

[B40] Muppirala U. K. Honavar V. G. Drena D. (2011). Predicting rna-protein interactions using only sequence information. BMC Bioinform. 12:489. 10.1186/1471-2105-12-489 22192482 PMC3322362

[B41] Peng L. H. Yin J. Zhou L. Q. Liu M. X. Zhao Y. (2018). Human microbe-disease association prediction based on adaptive boosting. Front. Microbiol. 9:2440. 10.3389/fmicb.2018.02440 30356751 PMC6189371

[B42] Qiu T. Han T. Y. Zhong L. X. Zhang Z. K. Chen G. (2014). Redundant correlation effect on personalized recommendation. Comput. Phys. Commun. 185, 489–494. 10.1016/j.cpc.2013.10.003

[B43] Sarah G. Jeff C. (2013). Rna in unexpected places: long non-coding rna functions in diverse cellular contexts. Nat. Rev. Mol. Cell Biol. 14, 699–712. 10.1038/nrm3679 24105322 PMC4852478

[B44] Shen C. Ding Y. J. Tang J. J. Guo F. (2019a). Multivariate information fusion with fast kernel learning to kernel ridge regression in predicting lncrna-protein interactions. Front. Genet. 9:716. 10.3389/fgene.2018.00716 30697228 PMC6340980

[B45] Shen C. Ding Y. J. Tang J. J. Jiang L. M. Guo F. (2019b). Lpi-ktaslp: prediction of lncrna-protein interaction by semi-supervised link learning with multivariate information. IEEE Access 7, 13486–13496. 10.1109/ACCESS.2019.2894225

[B46] Suresh V. Liu L. Adjeroh D. Zhou X. B. (2015). Rpi-pred: predicting ncrna-protein interaction using sequence and structural information. Nucleic Acids Res. 43, 1370–1379. 10.1093/nar/gkv020 25609700 PMC4330382

[B47] Tang X. Wang J. Zhong J. Pan Y. (2014). Predicting essential proteins based on weighted degree centrality. IEEE/ACM Trans. Comput. Biol. Bioinform. 11, 407–418. 10.1109/TCBB.2013.2295318 26355787

[B48] Ule J. Jensen K. Mele A. Darnell R. B. (2005). Clip: a method for identifying protein-rna interaction sites in living cells. Methods 37, 376–386. 10.1016/j.ymeth.2005.07.018 16314267

[B49] Ulitsky I. Bartel D. P. (2013). Lincrnas: genomics, evolution, and mechanisms. Cell 154, 26–46. 10.1016/j.cell.2013.06.020 23827673 PMC3924787

[B50] Wang F. J. Zheng Z. G. Guo J. F. Ding X. F. (2010). Correlation and quantitation of microrna aberrant expression in tissues and sera from patients with breast tumor. Gynecol. Oncol. 119, 586–593. 10.1016/j.ygyno.2010.07.021 20801493

[B51] Wang L. You Z. H. Chen X. Xia S. X. Liu F. Yan X.. (2017). A computational-based method for predicting drug-target interactions by using stacked autoencoder deep neural network. J. Comput. Biol. 25, 361–373. 10.1089/cmb.2017.0135 28891684

[B52] Wen Z. Qu Q. L. Zhang Y. Q. Wei W. Wen Z. Qu Q. L. (2017). The linear neighborhood propagation method for predicting long non-coding rna-protein interactions. Neurocomputing 273, 526–534. 10.1016/j.neucom.2017.07.065

[B53] Wu Z. R. Li W. H. Liu G. X. Tang Y. (2018). Network-based methods for prediction of drug-target interactions. Front. Pharmacol. 9:1134. 10.3389/fphar.2018.01134 30356768 PMC6189482

[B54] Xie C. Y. Yuan J. Li H. Li M. Zhao G. G. Bu D. C.. (2014). Noncodev4: exploring the world of long non-coding rna genes. Nucleic Acids Res. 42, 98–103. 10.1093/nar/gkt1222 24285305 PMC3965073

[B55] Xie D. Zhao Q. Liu H. S. Wang F. Yan G. Y. Chen X. (2018). Sscmda: spy and super cluster strategy for mirna-disease association prediction. Oncotarget 9, 1826–1842. 10.18632/oncotarget.22812 29416734 PMC5788602

[B56] Yuan J. Wu W. Xie C. Y. Zhao G. G. Zhao Y. Chen R. S. (2014). Npinter v2.0: an updated database of ncrna interactions. Nucleic Acids Res. 42, D104–D108. 10.1093/nar/gkt1057 24217916 PMC3965026

[B57] Zambelli F. Pavesi G. (2015). Rip-seq data analysis to determine rna-protein associations. Methods Mol. Biol. 1269, 293–303. 10.1007/978-1-4939-2291-8-18 25577386

[B58] Zeng X. X. Ding N. X. Rodríguezpatón A. Zou Q. (2017). Probability-based collaborative filtering model for predicting gene-disease associations. BMC Med. Genom. 10:76. 10.1186/s12920-017-0313-y 29297351 PMC5751590

[B59] Zhan Z. H. You Z. H. Li L. P. Zhou Y. Yi H. C. (2018). Accurate prediction of ncrna-protein interactions from the integration of sequence and evolutionary information. Front. Genet. 9:458. 10.3389/fgene.2018.00458 30349558 PMC6186793

[B60] Zhao Q. Liang D. Hu H. Ren G. F. Liu H. S. (2018a). Rwlpap: Random walk for lncrna-protein associations prediction. Protein Peptide Lett. 25, 830–837. 10.2174/0929866525666180905104904 30182833

[B61] Zhao Q. Yu H. F. Ming Z. Hu H. Ren G. F. Liu H. S. (2018b). The bipartite network projection-recommended algorithm for predicting long non-coding rna-protein interactions. Mol. Therapy Nucleic Acids 13, 464–471. 10.1016/j.omtn.2018.09.020 30388620 PMC6205413

[B62] Zhao Q. Zhang Y. Hu H. Ren G. F. Zhang W. Liu H. S. (2018c). Irwnrlpi: integrating random walk and neighborhood regularized logistic matrix factorization for lncrna-protein interaction prediction. Front. Genet. 9:239. 10.3389/fgene.2018.00239 30023002 PMC6040094

[B63] Zheng X. X. Wang Y. Tian K. Zhou J. G. Guan J. H. Luo L.. (2017). Fusing multiple protein-protein similarity networks to effectively predict lncrna-protein interactions. BMC Bioinform. 18:420. 10.1186/s12859-017-1819-1 29072138 PMC5657051

[B64] Zhou T. Su R. Q. Liu R. R. Jiang L. L. Wang B. H. Zhang Y. C. (2009). Ultra accurate personalized recommendation via eliminating redundant correlations. Phys. Soc. arXiv:0805.4127. 10.1088/1367-2630/11/12/123008

[B65] Zhu L. C. Su F. C. Xu Y. C. Zou Q. (2018). Network-based method for mining novel hpv infection related genes using random walk with restart algorithm. BBA Mol. Basis Dis. 1864, 2376–2383. 10.1016/j.bbadis.2017.11.021 29197659

[B66] Zou Q. Qu K. Y. Luo Y. M. Yin D. H. Ju Y. Tang H. (2018). Predicting diabetes mellitus with machine learning techniques. Front. Genet. 9:515. 10.3389/fgene.2018.00515 30459809 PMC6232260

